# Tumor necrosis factor-α attenuates starvation-induced apoptosis through upregulation of ferritin heavy chain in hepatocellular carcinoma cells

**DOI:** 10.1186/1471-2407-13-438

**Published:** 2013-09-25

**Authors:** Xingrui Kou, Yingying Jing, Weijie Deng, Kai Sun, Zhipeng Han, Fei Ye, Guofeng Yu, Qingmin Fan, Lu Gao, Qiudong Zhao, Xue Zhao, Rong Li, Lixin Wei, Mengchao Wu

**Affiliations:** 1 Tumor Immunology and Gene Therapy Center, Eastern Hepatobiliary Surgery Hospital, Second Military Medical University, 225 Changhai Road, Shanghai 200438, P. R China; 2 Medical Sciences Research Center, renji hospital, school of medicine, Shanghai jiaotong University, Shanghai, PR. China

**Keywords:** TNF-α, Starvation, NF-κB, Ferritin heavy chain, Autophagy, Hepatocellular carcinoma

## Abstract

**Background:**

Tumor microenviroment is characteristic of inflammation, ischemia and starvation of nutrient. TNF-α, which is an extraordinarily pleiotropic cytokine, could be an endogenous tumor promoter in some tumor types. The basic objective of this study was to investigate the effects of TNF-α on the cell viability and apoptosis of hepatocellular carcinoma cells under serum starvation, and to identify the molecular mechanisms involved.

**Methods:**

For this purpose, five different concentrations of TNF-α and two different serum settings (serum-cultured and serum-deprived) were used to investigate the effects of TNF-α on the cell viability and apoptosis of Hep3B and SMMC-7721 cells.

**Results:**

TNF-α (10 ng/ml) attenuated serum starvation-induced apoptosis of hepatocellular carcinoma cells, and autophagy conferred this process. BAY11-7082, a specific inhibitor of NF-κB, reversed the suppression of serum starvation-induced apoptosis by TNF-α. Moreover, TNF-α-induced NF-κB transactivation was suppressed by autophagy inhibitor 3-MA. In addition, TNF-α up-regulated Ferritin heavy chain (FHC) transiently by NF-κB activation and FHC levels were correlated with the TNF-α-induced protection against serum starvation-mediated apoptosis of hepatocellular carcinoma cells. Furthermore, FHC-mediated inhibition of apoptosis depended on suppressing ROS accumulation.

**Conclusions:**

Our findings suggested that autophagy conferred the TNF-α protection against serum starvation-mediated apoptosis of hepatocellular carcinoma cells, the mechanism involved with the activation of the TNF-α/ NF-κB /FHC signaling pathway.

## Background

Many experimental evidence indicates that TNF-α is associated with the survival of cancer cells [1,2]. TNF-α-mediated the killing of certain cancer cells has been demonstrated [3,4]. Although TNF-α itself was named for its ability to induce cell death, it has been known that TNF-α stimulation also can induce activation of the transcription factor NF-κB [5-8]. Many normal cells are not killed by TNF-α and this may be related to NF-κB transactivation; blockade of NF-κB sensitizes cells to TNF-α and augments induced apoptotic cell death [9]. TNF-α induced NF-κB transactivation through the pathway of IκB kinase complex phosphorylation, degradation of IκBα and release of cytoplasm-sequestered [10]. TNF-α-induced NF-κB transactivation is mainly composed of a hetero-dimer of p65 (RelA) and a p50 subunits. NF-κB transactivation can activate expression of a wide variety of genes including the Ferritin heavy chain [11,12]. Recent studies have shown that NF-κB-regulated FHC can inhibit caspase activity and can prevent TNF-α-induced apoptosis [13]. Additional studies have shown that suppression of IAP genes sensitized endothelial cells to TNF-α-induced apoptosis. We have previously shown that Hep3B and SMMC-7721 cells are resistant to serum starvation-induced cell death due to activation of NF-κB by TNF-α. In the present study, we show that serum starvation induced significant apoptosis in the Hep3B and SMMC-7721 cells, and this cell death was attenuated by pre-incubation of TNF-α via suppression of caspase activation and coincident with Ferritin heavy chain up-regulation. Inhibition of NF-κB transactivation using a pharmacological inhibitor of IKK abrogated the TNF-α-induced protection against serum starvation killing. We demonstrate that temporal TNF-α-mediated suppression of serum starvation-mediated apoptosis may be due to the transient up-regulation of FHC by TNF-α.

## Methods

### Cell culture and regent

Human hepatocellular carcinoma cell lines Hep3B and SMMC-7721 were purchased from Cell Bank of Type Culture Collection of Chinese Academy of Sciences, Shanghai Institute of Cell Biology, Chinese Academy of Sciences. Human hepatocellular carcinoma cell lines Hep3B and SMMC-7721 were cultured at 37°C, with 5% CO_2_, in Dulbecco’s modified Eagle’s medium (high glucose) (GIBCO, Invitrogen) with 10% fetal bovine serum, supplemented with 2 mM L-glutamine, 100 U/ml penicillin, and 100ug/ml streptomycin. Cells were subcultured every 3 days when they reached 70%-80% confluence. 3-Methyladenine (3-MA, Sigma-Aldrich) was dissolved in heated sterile double distilled water to make a 400 mM stock solution and then added to the medium after heating for a final concentration of 5 mM.

### Western blot analysis

Total protein was extracted from cells using lysis buffer and the protein concentrations were measured by BCA protein assay. The cell lysates were loaded on SDS-PAGE, electrophoresed and transferred onto the PVDF membranes. The membranes were blocked in 5% non-fat dry milk in 0.01% Tween/PBS, incubated in primary antibody overnight at 4°C, then incubated in HRP-conjugated secondary antibodies and developed using ECL plus detection reagent. The primary antibodies used in this study are: P62 (#5114, Cell Signaling Technology), LC3 (#4108, Cell Signaling Technology), IκBα (#4812, Cell Signaling Technology), P65 (sc-8008, Santa Cruz Biotechnology, Inc), Cleaved caspase-3 (ab52293, Abcam), Caspase-8 (AP0358, Bioworld), FHC (ab65080, Abcam).

### MTT assay

3-(4,5-dimethylthiazol-2-Yl)-2,5-diphenyltetrazolium bromide assay (Sigma Aldrich) was used to determine cell survival. Cell count was adjusted to 1 × 10^4^ cells/ml 100 μl of cells suspension was plated in each well of 96-well plate. At the end of the various treatment, the medium was removed and cells were immediately washed with PBS, then 150 μl/well of MTT solution was added. After 3 h, media containing MTT was removed and 100 μl of DMSO was added to each well to dissolve the formazan crystals. Absorbance was taken at 570 nm and 655 nm. Experiments were performed in triplicate and repeated three times.

### RNA isolation and real-time PCR

Total cellular RNA was isolated from SMCs using an RNeasy Mini Kit (Qiagen) according to the manufacturer’s instructions. RNA was subjected to reverse transcription using Taqman reverse transcription kit (Applied Biosystems) following the manufacturer’s instructions. Real time PCR amplifications were performed using iQTM SYBR Green supermix (BIO-RAD). The relative quantities of mRNAs were obtained by using the comparative Ct method and were normalized with glyceraldehydes-3-phosphate dehydrogenase (GAPDH). Primers sequences for FHC were: forward 5′-ATTTCCCCATAGCCGTG -3′, reverse 5′-GCCTGGATAGATTTCTGATTC -3′.

### The measurement of ROS accumulation

The intracellular ROS levels were detected by means of an oxidation-sensitive fluorescent probe (DCFH-DA). Briefly, the cells were cultured and treated with the indicated time intervals. Then, the cells were harvested, washed twice with PBS, incubated with DCFH-DA (1 μM) in serum-free DMEM at 37°C in a 5% CO2 incubator for 20 minutes, washed twice with PBS and analyzed by Immunofluorescence microscope.

### Transient transfection and identification of autophagy

Hep3B and SMMC-7721 cells were seeded (5 × 10^4^ cells/well) in 96-well plates for overnight, then GFP-LC3 expressing plasmids were transiently transfected into the cells using Fugene HD transfection reagent (Roche) according to the manu-facturer‘s instructions. After cultured for 24 h to ensure the expression of GFP-LC3, the cells were subjected to different treatment. At the end of the treatment, autophagy was detected by counting the percentage of cells with GFP-LC3-positive dots under fluorescence microscope. Aminimum of 200 cells per sample was counted in triplicate for each experiment.

### Plasmid transfection

The site-specific, signal-induced degradation of IκBα depends on phosphorylation at Ser 32 and 36. Therefore, the pBαbe-SR-IκBα plasmid that consisted of a double point mutation (Ser to Lactamine) was thus resistant to phosphorylation. The mutant and control plasmids were transiently transfected into Hep3B and SMMC-7721 cells by Lipofectamine. Hep3B and SMMC-7721 cells were removed by trypsin/EDTA treatment and seeded at a density of 2x10^5^ cells/ml in 6-cm culture dishes. Cells were grown to 90% confluence and subjected to 24-h synchronization in serum-free medium. Hep3B and SMMC-7721 cells were transfected with 4 μg of the pBαbe-SR-IκBα or control pBαbe plasmid per dish with the use of Lipofectamine. After incubation for 6 h, the transfection medium was replaced by fresh medium for an additional 48-h incubation to allow for gene expression to occur.

### Short hairpin RNA

shRNA candidate target sequences to Beclin1 is 5′-GCAGATGAGGAAGATCGCCTT-3′. The oligonucleotides encoding the shRNA sequence were inserted into the GFP express vector pGCL-GFP (Shanghai GeneChem, shanghai). SCR-shRNA was used as a negative RNAi control. The recombinant virus was packaged using Lentivector Expression Systems (Shanghai GeneChem).

### Apoptosis assays

Hep3B and SMMC-7721 cells were plated (1 × 10^6^ cells/well) onto 6-well plates for overnight in incubator to resume exponential growth. At the end of the various treatment, every sample were removed from the medium and washed with PBS twice. Then the cells were stained with FITC-conjugated Annexin V and propidium iodide (PI), using Annexin V-FITC Apoptosis Detection kit and according to manufacturer‘s recommendation (Calbiochem). Flow cytometry (BD Biosciences, USA) was used to determine the percentage of apoptotic cells.

### NF-κB-dependent reporter gene assay

NF-κB luciferase reporter assays were performed as described previously [14]. Briefly, cells were co-transfected with a pNF-κB-luc reporter construct and a renilla luciferase-expressing plasmid (internal control to normalize for transfection efficiency) using Lipofectamine 2000 according to the manufacturer’s instructions. At the end of the various treatment, firefly and renilla luciferase activities were assessed using a dual luciferase reporter gene assay kit. NF-κB transcriptional activity = relative light units of firefly luciferase/relative light units of renilla luciferase.

### Immunofluorescence

Immunofluorescence staining was performed according to standard protocol (Santa Cruz Biotechnology). Hep3B and SMMC7721 cells were seeded (1 × 10^5^ cells/well) on a 48-well plate, cultured in DMEM without FBS and Antibiotic for 6 h, and 3-MA was added to the cell culture at the same time. Then cells were treated with or without TNF-α (10 ng/ml) for 24 h, then the cells were washed twice with PBS, and fixed in 4% paraformaldehyde and 0.1% Triton X 100 in PBS buffer at 4°C for 30 minutes. After being washed with PBS, the cells were incubated with the blocking solution (10% goat serum in PBS), and then incubated overnight with the primary antibodies, washed with PBS, and finally incubated with secondary antibodies at 37°C for 2 hours. After being stained with DAPI, all matched samples were photographed using an immunofluorescence microscope and identical exposure times.

### Statistical analysis

All the experiments were performed at least three times. Student’s t-test was used for all the statistical analyses, and the differences were considered significant if the p value was less than 0.05.

## Results

### TNF-α attenuated serum starvation-induced apoptosis in Hep3B and SMMC-7721 cells

**Figure 1:**
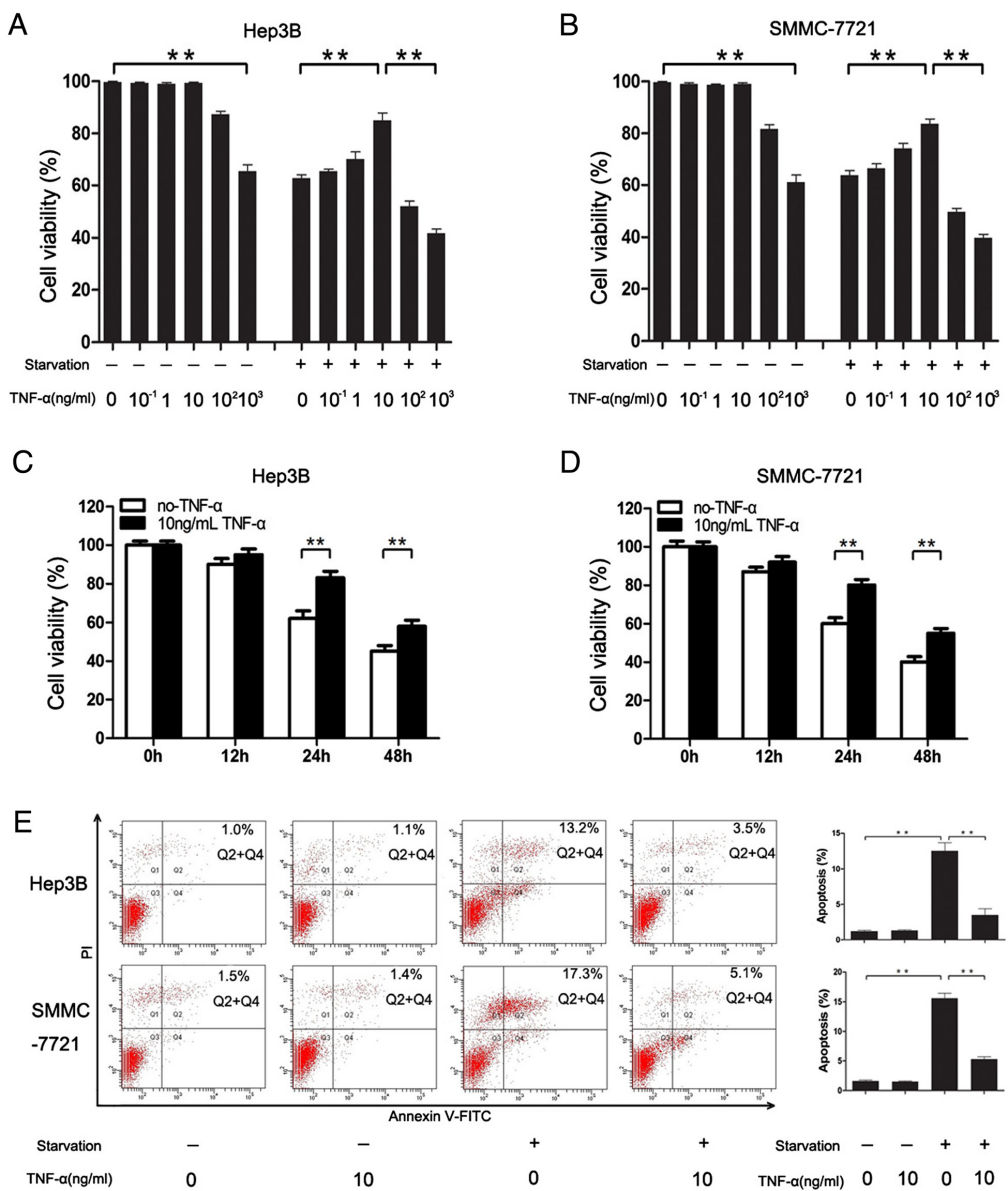
**TNF-α attenuated serum starvation-induced apoptosis in Hep3B and SMMC-7721 cells. (A**, **B**, **C** and **D)** Cell activity were detected by MTT analysis. Hep3B **(A)** and SMMC-7721 **(B)** cells were treated with or without 6 h pre-treatment of serum starvation (that is nutrient- and serum-free medium), then treated with TNF-α (0, 0.1, 1, 10, 100, 1000 ng/ml) for 24 h. Hep3B **(C)** and SMMC-7721 **(D)** cells were treated with 10 ng/ml TNF-α for 12, 24 and 48 h with 6 h pre-treatment of serum starvation. **(E)** Cells were cultured in medium with or without 10% FBS for 6 h, then treated with or without 10 ng/ml TNF-α for 24 h. Flow cytometry was used to test cell apoptosis rate. Data represent the mean ± SEM of three independent experiments. **P < 0.01; Student’s t-test.

Five different concentrations of TNF-α and two different serum settings (serum-cultured and serum-deprived) were used to investigate the effects of TNF-α on the cell viability and apoptosis of Hep3B and SMMC-7721 cells. We performed a cell viability assay, TNF-α did not significantly affect the cell viability of serum-cultured Hep3B and SMMC-7721 cells up to 100 ng/ml, which significantly suppressed the cell viability of serum-cultured and serum-deprived cells. Interestingly, low-dose TNF-α (0.1, 1 and 10 ng/ml) prevented the loss of cell viability of serum-deprived cells, especially in the 10 ng/ml TNF-α group (Figure 1A and 1B). This concentration was therefore used to evaluate the effect of TNF-α on serum starvation-induced cell death of Hep3B and SMMC-7721 cells. Following 6 hour ‘pre-starvation’, cells were incubated with TNF-α for 0 h to 48 h and cell viability was detected by MTT. At 0 h and 12 h, there was no significant difference between the cell viability of serum starvation + TNF-α group and that of serum starvation group; at 24 h and 48 h, the cell viability of serum starvation + TNF-α group was significantly higher than that of serum starvation group (Figure 1C and 1D). Flow cytometry analysis revealed that the percentage of apoptosis Hep3B and SMMC-7721 cells in the serum starvation + TNF-α group significantly decreased (3.51% ± 1.21% vs 12.12% ± 1.42% ; 4.88% ± 1.02% vs 17.33% ± 1.31%, P < 0.01) compared with the serum starvation group; there were no significant differences between the control and TNF-α groups of serum-cultured Hep3B and SMMC-7721 cells (1.16% ± 0.54% vs 1.02% ± 0.45%; 1.46% ± 0.64% vs 1.53% ± 0.65%, P > 0.05, Figure 1E). These results indicate that TNF-α attenuates serum starvation-induced apoptosis in Hep3B and SMMC-7721 cells.

### 3-Methyladenine (3-MA) attenuated TNF-α protection against serum starvation-mediated apoptosis

**Figure 2:**
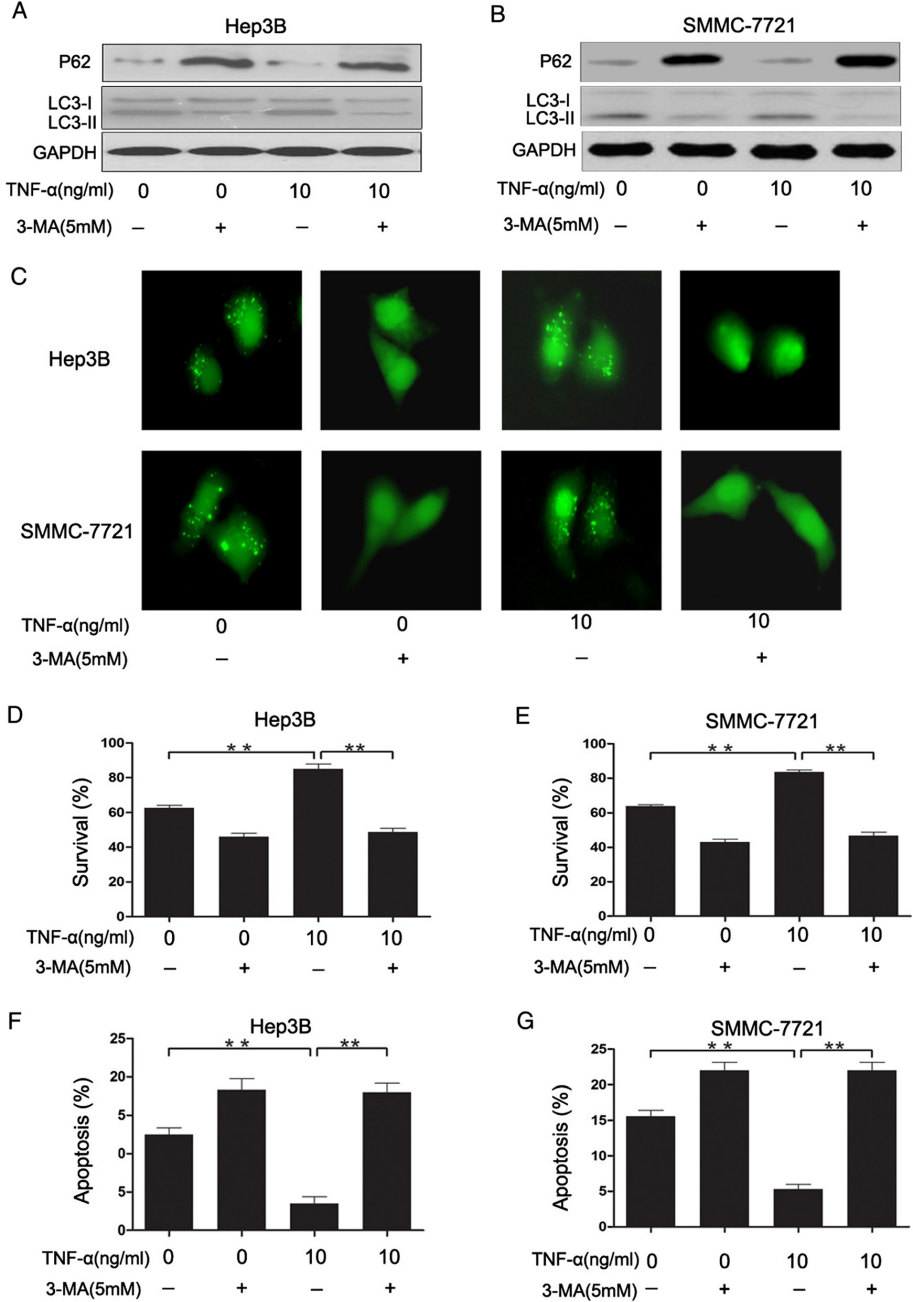
**3-MA attenuated TNF-α protection against serum starvation-mediated apoptosis.** Hep3B and SMMC-7721 cells were cultured under serum starvation condition for 6 h, and 3-MA was added to the cell culture at the same time. Then cells were treated with or without TNF-α (10 ng/ml) for 24 h. **(A** and **B)** Total cellular protein (50 μg) was separated by SDS-PAGE and immunoblotted for P62, LC3 and GAPDH. **(C)** Cells were transfected with GFP-LC3 for 24 h before they were treated with serum starvation. At the end of treatment, the cells were examined by fluorescence microscopy. **(D** and **E)** Cell activity was determined by MTT analysis. **(F** and **G)** Apoptosis was measured by flow cytometry. Data are presented as the mean ± SEM from three independent experiments. **P < 0.01; Student’s t-test.

To investigate whether autophagy signaling pathway was related to the effect of TNF-α, its inhibitor 3-MA administered prior to TNF-α treatment. Western blotting analysis showed that serum starvation resulted in an increase in LC3II and a decrease in P62, however, the treatment of 3-methyladenine reversed the change (Figure 2A and 2B). Meanwhile, 3-MA reduced the GFP-LC3 dot aggregation, as well as the LC3II protein on western blotting (Figure 2C). After treatment of TNF-α, the cell viability of serum starvation + TNF-α group was significantly higher than that of serum starvation group. However, when the cells were grown in the presence of TNF-α and 3-MA, 3-MA blocked the effect produced by TNF-α. There were no significant differences between the 3-MA and 3-MA + TNF-α groups of serum-deprived cells (Figure 2D and 2E). To further confirm the results from our MTT data, we used Annexin V-PI staining. Flow cytometry analysis showed that treatment with TNF-α decreased the population of apoptotic cells, while treatment with TNF-α + 3-MA increased the population of apoptotic cells. There were no significant differences between the 3-MA and 3-MA + TNF-α groups (Figure 2F and 2G). In addition, we inhibited autophagy by shRNAs to Beclin1, and obtained similar results with 3-MA (Additional file 1: Figure S1). These results demonstrate that autophagy conferred the TNF-α protection against serum starvation-mediated apoptosis of hepatocellular carcinoma cells.

### 3-MA suppressed TNF-α-induced NF-κB transactivation

**Figure 3:**
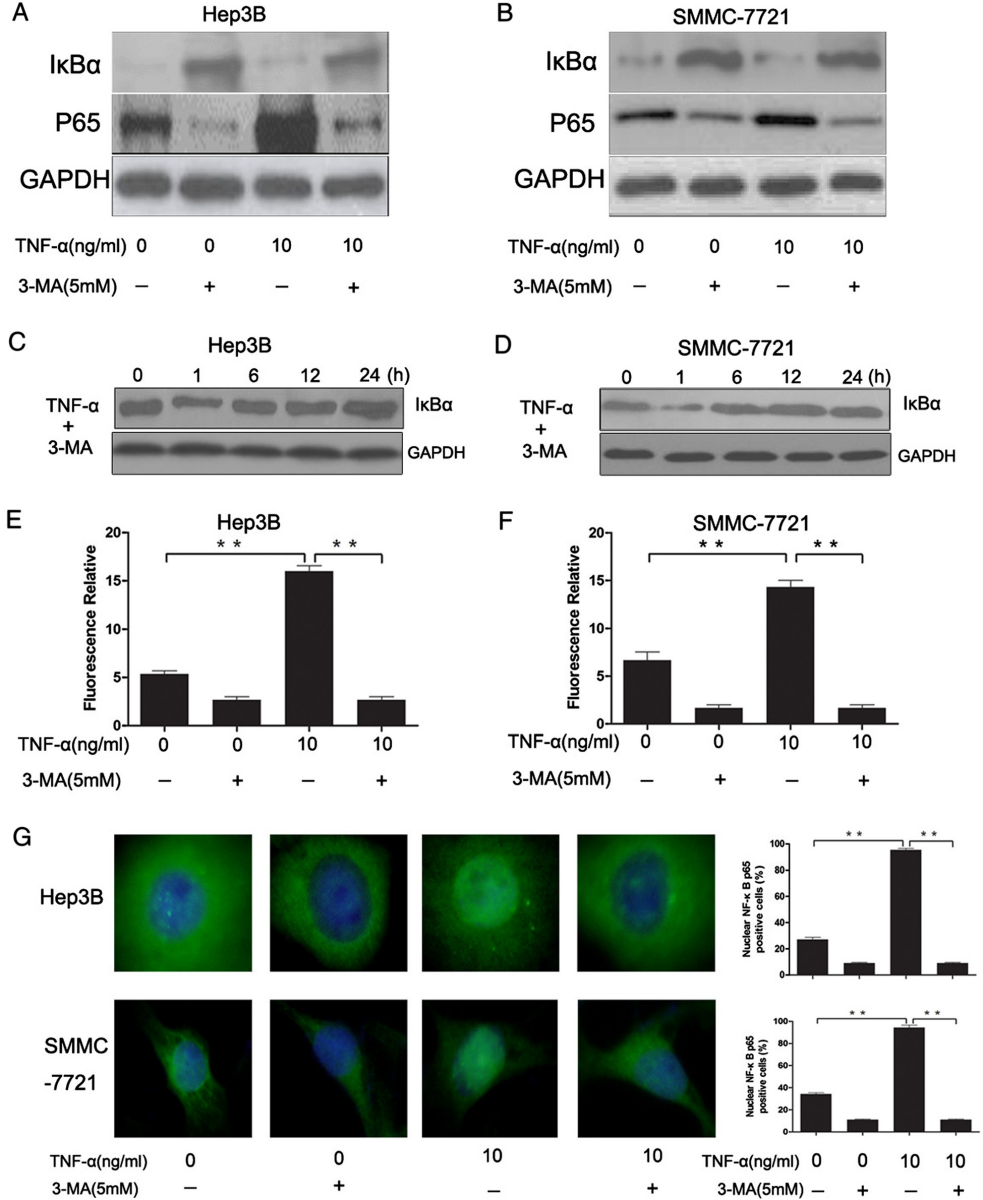
**TNF-α induced NF-κB activation and 3-MA attenuated the activation of NF-κB.** Hep3B and SMMC-7721 cells were cultured under serum starvation condition for 6 h, and 3-MA was added to the cell culture at the same time. Then cells were treated with or without TNF-α (10 ng/ml) for 24 h. **(A** and **B)** Total cellular protein(50 μg) was separated by SDS-PAGE and immunoblotted for IκBα, P65 and GAPDH. **(C** and **D)**: Hep3B and SMMC-7721 cells were stimulated with TNF-α in the presence of 3-MA for the indicated times (hours). Western boltting was performed with anti-IκBα. **(E** and **F)** Cells were transduced using an NF-κB luciferase construct, then the cells were treated according to the previously described steps. At the end of the various treatment, firefly and renilla luciferase activities were assessed using a dual luciferase reporter gene assay kit. **(G)** NF-κB nuclear translocation was assessed by immunofluorescence staining for NF-κB p65 (green), cell nuclei were detected by DAPI (× 400). The percentage of the p65 nuclei-positively stained cells to the total cells was quantified. Data are presented as the mean ± SEM from three independent experiments. **P < 0.01; Student’s t-test.

Previous studies have shown that NF-κB is a powerful transcription factor that blocks apoptosis [15]. TNF-α can induce NF-κB transactivation via IκB kinase (IKK) complex phosp orylation, which lead to degradation of IκBs and the consequent translocation of NF-κB to nucleus [9,10,16,17]. We examined TNF-α mediated NF-κB transactivation in Hep3B and SMMC-7721 cells. The expression levels of representative upstream and downstream signaling proteins involved in NF-κB activation were detected by Western blotting analysis. Hep3B and SMMC-7721 cells were cultured under serum starvation condition in the presence or absence of 3-MA for 6 h, then cells were treated with or without TNF-α (10 ng/ml) for 24 h. After treatment of TNF-α, a significant increase protein expressions of NF-κB p65 was observed, while TNF-α decreased protein expressions of IκBα. Interestingly, 3-MA reversed the effect of TNF-α (Figure 3A and 3B). Hep3B and SMMC-7721 cells were stimulated with TNF-α in the presence of 3-MA for the indicated times (hours). It showed that TNF-α resulted in the rapid loss of IκBα expression in cells, which was followed by its re-expression 1 hour later (Figure 3C and 3D). The rapid activation may be attributed to the control of proteasome as described in the article [18]. To further confirm the results from the western blotting, we performed a NF-κB-dependent reporter gene assay. It showed that TNF-α treatment resulted in a significant increase of luciferase activity in Hep3B and SMMC-7721 cells, while 3-MA reversed this change (Figure 3E and 3F). In addition, NF-κB nuclear translocation was assessed by immunofluorescence staining for NF-κB p65. The green nuclear signal was an indication of the activation. TNF-α significantly increased p65 subunit translocation, however, 3-MA suppressed the translocation of p65 subunit (Figure 3G). These results showed that 3-MA significantly suppressed NF-κB activity induced by TNF-α (P < 0.05), which indicated that autophagy conferred the TNF-α-induced NF-κB activation.

### NF-κB inhibitor BAY11-7082 inhibited TNF-α protection against serum starvation-mediated apoptosis

**Figure 4:**
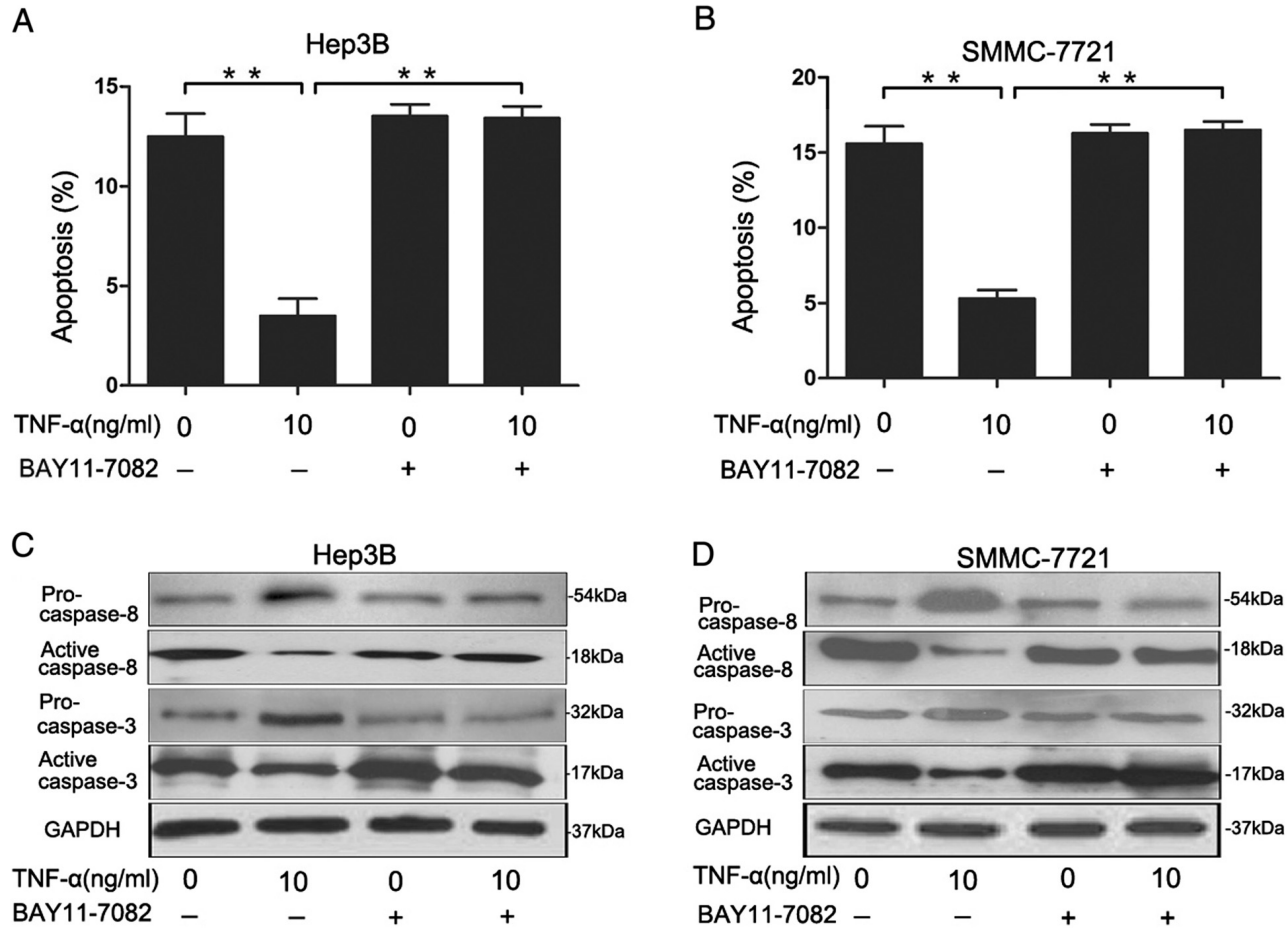
**NF-κB inhibitor BAY11-7082 decreased TNF-α protection against serum starvation-mediated apoptosis.** Serum-deprived Hep3B and SMMC-7721 cells were cultured with TNF-α (10 ng/ml) and/or BAY11-7082 (10 μM). **(A** and **B)** Cells were harvested at 24 h, FITC/PI staining was performed to detect the apoptosis. Data are presented as the mean ± SEM from three independent experiments. **P < 0.01; Student’s t-test. **(C** and **D)** Expression of caspase-8 and cleaved caspase-3 were determined by Western blotting.

Because serum starvation-induced apoptosis was inhibited by TNF-α, which enhanced the transactivation of NF-κB, it suggested a link between NF-κB transactivation and inhibition against serum starvation-induced apoptosis. To determine whether NF-κB transactivation is important in TNF-α protection against serum starvation-induced apoptosis, we used BAY11-7082 to inhibit NF-κB transactivation in Hep3B and SMMC-7721 cells. After treatment of TNF-α with BAY11-7082, the percentage of apoptosis cells significantly increased compared with the TNF-α group; there were no significant differences between the BAY11-7082 group and TNF-α + BAY11-7082 group (Figure 4A and 4B). Moreover, western blotting showed that TNF-α inhibited expression of caspase-8 and cleaved caspase-3, but these were reversed by BAY11-7082 (Figure 4C and 4D). Furthermore, the mutant plasmids were transiently transfected into Hep3B and SMMC-7721 cells by Lipofectamine to inhibit the activation of NF-κB, and obtained similar results with BAY11-7082 (Additional file 1: Figure S2). These results indicated that TNF-α prevented Hep3B and SMMC-7721 cells from serum starvation-induced apoptosis via transactivation of NF-κB.

### Overexpression of FHC induced by TNF-α inhibited apoptosis signaling in serum-deprived cells

**Figure 5:**
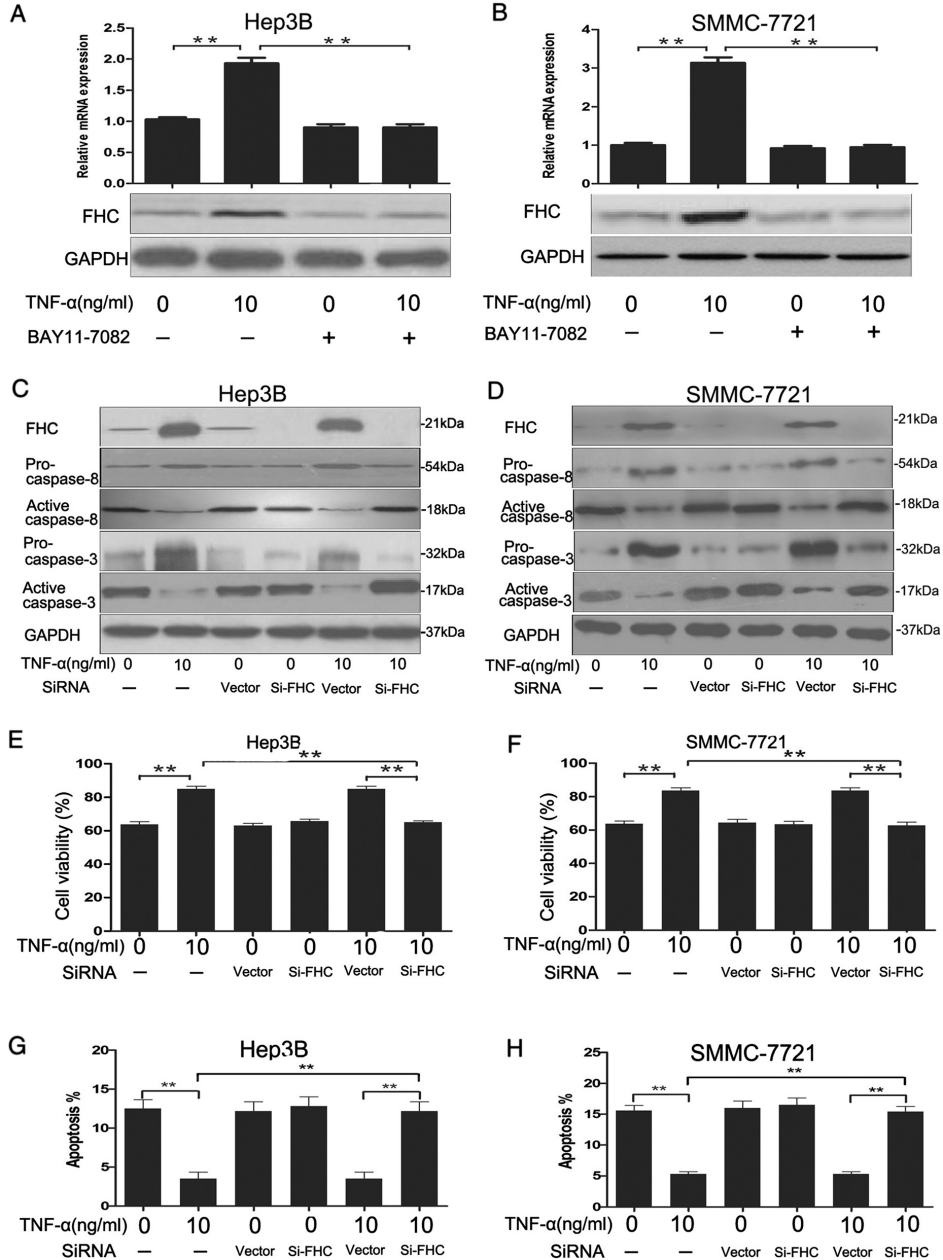
**Overexpression of FHC induced by TNF-α inhibited apoptosis signaling in serum-deprived cells. (A** and **B)** Serum-deprived Hep3B and SMMC-7721 cells were cultured with or without TNF-α (10 ng/ml) and/or BAY11-7082 (10 μM). The expression of FHC was determined by RT-PCR and Western-blotting analysis. Hep3B **(C)** and SMMC-7721 **(D)** cells were transfected with siFHC or Vector and then serum-deprived cells were treated with or without TNF-α (10 ng/ml) for 24 h. Western blotting was performed with anti-FHC or anti-caspase-8 or anti-cleaved caspase-3 or GAPDH. **(E** and **F)** Cell viability was detected by MTT assay. **(G** and **H)** Cells were harvested and stained with Annexin-V-FITC and PI, apoptosis was measured by flow cytometry. Data are presented as the mean ± SEM from three independent experiments. **P < 0.01; Student’s t-test.

It has been known NF-κB can regulate the expression of the anti-apoptotic gene products, including IAPs [19,20], Bcl-2 [21], Bcl-xL [22-24], Mcl-1, TRAF-1 [25], Survivin and FHC [26]. FHC is upregulated by TNF-α through activation of NF-κB, is essential to inhibit apoptosis in NF-κB null cells [26]. Western blotting and RT-PCR analysis showed that TNF-α treatment increased the expression of FHC compared with control group, this was inhibited by BAY11-7082 (Figure 5A and 5B). To determine whether the induction of FHC by NF-κB serves a protective function, we blocked FHC expression in the cells by small interfering RNA (siRNA). After treatment of TNF-α, the expression of caspase-8 and cleaved caspase-3 in cells transfected with siFHC was increased compared with that transfected with vector siRNA (Figure 5C and 5D). Consistent with the results obtained by western blotting, cell viability of TNF-α + siFHC group was reduced compared with TNF-α + vector group (Figure 5E and 5F). And, fluorescence microscopy showed that the population of apoptotic cells was decreased by TNF-α treatment, while this was inhibited by TNF-α + siFHC treatment (Figure 5G and 5H). These results suggested that the induction of FHC by NF-κB is required to suppress serum starvation-induced apoptosis.

### ROS inhibition by FHC protected cells from serum starvation-induced apoptosis

**Figure 6:**
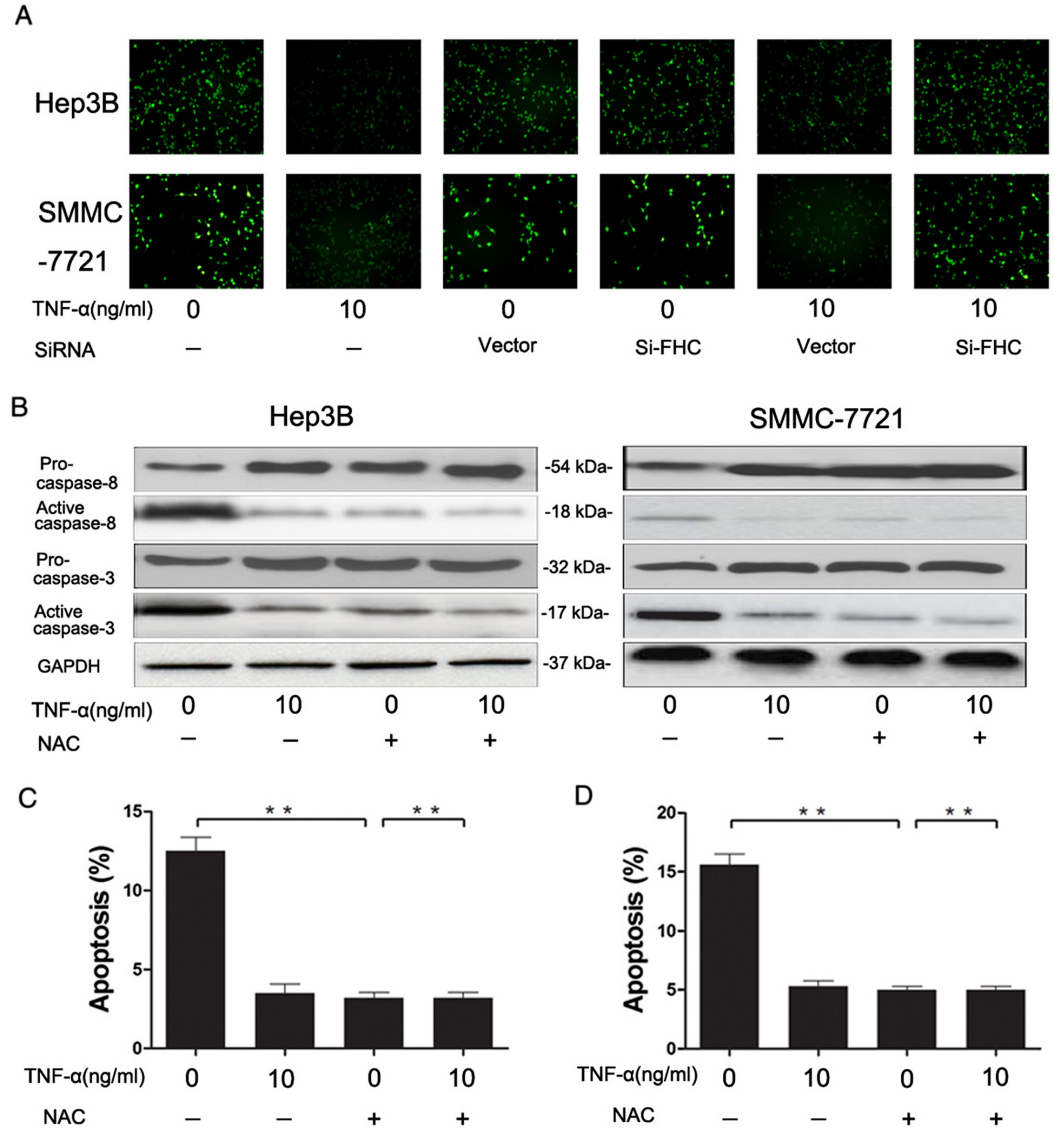
**ROS inhibition by FHC protected cells from serum starvation-induced apoptosis.** Hep3B and SMMC-7721 cells were transfected with siFHC or Vector and then serum-deprived cells were treated with or without TNF-α (10 ng/ml) for 24 h. **(A)** The formation of reactive oxygen species (ROS) was assayed by measuring the fluorescence of dichlorofluorescein (DCF). **(B)** Serum-deprived Hep3B and SMMC-7721 cells were cultured with or without TNF-α (10 ng/ml) and/or N-acetylcysteine (NAC) (10 mM). The expression of pro/active-caspase-8 and pro/active- caspase-3 were determined by Western-blotting analysis. **(C)** and **(D)**: Serum-deprived Hep3B and SMMC-7721 cells were cultured with or without TNF-α (10 ng/ml) and/or N-acetylcysteine (NAC) (10 mM).Flow cytometry was used to test cell apoptosis rate. Data are presented as the mean ± SEM from three independent experiments. **P < 0.01; Student’s t-test.

ROS plays an important role in the induction of apoptosis by serum starvation. To determine whether the induction of FHC inhibited serum starvation-induced apoptosis via ROS inhibition, we used siRNA to block FHC expression. DCF fluorescence showed that TNF-α significantly decreased the level of intracellular ROS, however, siFHC treatment reversed this change (Figure 6A). Treatment with NAC (a ROS scavenger) suppressed TNF-α-induced caspase cascade inhibition (Figure 6B). These results indicated that FHC inhibited serum starvation-induced apoptosis via ROS inhibition.

## Discussion

TNF-α is an extraordinarily pleiotropic cytokine produced mainly by activated macrophages and a few by several other types of cell [27,28]_._ Under normal physiological conditions TNF-α plays a part in maintenance and homeostasis of host defence and the immune system; on the other side, its “inappropriate” overexpression is implicated in malignant disease and pathological injury, such as insulin resistance, autoimmunity, septic shock, allergy and allograft rejection [28,29]. TNF-α is well known for the anticancer properties and is being an effective anticancer agent for the treatment of patients with locally advanced solid tumors [29]. However, TNF-α is a double-edged sword for cancer. TNF-α could be an endogenous tumor promoter in tumor microenvironment, because TNF-α stimulates cancer cells’ growth, proliferation, invasion and metastasis, and tumor angiogenesis [30-32]. It is of significance to research the relationship between TNF-α and HCC, which is one of the 10 most common human carcinomas in the world. Past studies have shown that TNF-α contributes to liver carcinogenesis early in the preneoplastic phase through driving oval cell proliferation [33]. In the model of p-glyco-protein 2 (Mdr2)-knockout mice, TNF-α and the activation of NF-кB accelerated the process of tumor in the occurrence of HCC [34]. In our study, it is consistent with previous studies [4,29], high doses of TNF-α (100 or 1000 ng/ml) enhanced serum starvation-induced apoptosis, which maybe due to the direct killing effect of TNF-α on tumor cells. Interestingly, low doses of TNF-α (0.1, 1 and 10 ng/ml) attenuated serum starvation-induced apoptosis, especially in the 10 ng/ml TNF-α group (Figure 1). The results suggested that TNF-a may play an important role in HCC survival.

In the present study, we examine the TNF-α effect on serum starvation-mediated apoptosis of HCC cells in light of the potential importance of TNF-α in HCC survival, we examine whether the TNF-α effect on serum starvation-induced apoptosis of HCC cells via autophagy. Autophagy can also be considered a temporary survival mechanism during periods of serum starvation where self-digestion provides an alternative energy source and also may facilitate the disposal of unfolded proteins under stress conditions [35]. Autophagy has a dynamic role in cancer—both as a tumor suppressor early in progression and later as a protumorigenic process, critical for tumor maintenance and therapeutic resistance [36-38]. Evolving tumors develop regions of hypoxia and nutrient limitation, where elevated autophagy activation has been found previously to promote tumor survival. Moreover, the role for the TNF-α-induced NF-κB pathway in autophagy was supported by the evidence that the IKK complex was necessary for the stimulation of autophagy by several factors [18,39,40]. Therefore, it is necessary to examine whether the TNF-α effect on serum starvation-induced apoptosis of HCC cells via autophagy. In the present study, we showed that 3-Methyladenine (3-MA) attenuated TNF-α protection against serum starvation-mediated apoptosis (Figure 2). This suggested that autophagy conferred the TNF-α protection against serum starvation-mediated apoptosis.

In our study, Pre-induction of cells with the pharmacological inhibitor-Bay11-7082, inhibited NF-κB transactivation, attenuated TNF-α protection against serum starvation-mediated apoptosis in Hep3B and SMMC-7721 cells. Our results suggested that TNF-α induced transactivation of NF-κB in favor of survival or anti-apoptotic signaling in Hep3B and SMMC-7721 cells (Figure 3 and Figure 4). Ferritin heavy chain (FHC) is one of the NF-κB-regulated genes that counteract apoptotic signaling by TNF-α in a number of cells [26]. It is identified that FHC as a pivotal effector of the antioxidant and protective actions of NF-κB downstream of TNF-Rs [41]. FHC is upregulated by TNF-α through a mechanism controlled by NF-κB, which is required for inhibition of TNF-α-induced killing and blocking PCD in NF-κB-deficient cells [26]. FHC is one of several acute-phase proteins, induced in the liver during the organismal response to stress, injury and infection. FHC might also play a prominent role in NF-κB-dependent oncogenesis, tumor progression and cancer chemo- and radio-resistance. High levels of FHC have in fact been found in several tumors and have been associated with resistance to anti-cancer treatment and an aggressive malignant phenotype [42]. In our Hep3B and SMMC-7721 cells, FHC was present at very low detectable level endogenously, but increased significantly 24 h after TNF-α treatment. The kinetics of TNF-α-induced FHC expression was directly correlated with kinetics of TNF-α protection against serum starvation-induced apoptosis in Hep3B and SMMC-7721 cells. In order to confirm that FHC contributes to apoptosis resistance in Hep3B and SMMC-7721 cells, we suppressed FHC expression in the cells by small interfering RNA (siRNA) and assayed their apoptosis sensitivity. Apoptotic cell population in Hep3B and SMMC-7721 cells transfected with FHC siRNA was larger than that transfected with control siRNA at concentration of 10 ng/ml TNF-α exposure (Figure 5). These results suggested that FHC prevents apoptosis induced by serum starvation.

The observation that FHC protected HCC cells from serum starvation-induced apoptosis prompted us to investigate whether FHC mediated the inhibition of ROS by serum starvation. Human mesothelial cells stably over-expressing FHC generated less H_2_O_2_ when challenged by asbestos and were resistant to apoptosis induced by oxidant stimuli compared with control cells [43]. It suggested FHC reduced intracellular oxidative stress triggered by asbestos exposure in mesothelial cells and contributes to apoptosis resistance by diminishing ROS generation. Our results are consistent with the results obtained by others, using Hep3B and SMMC-7721 cells. Recently, it is reported the key role of FHC in regulating apoptosis during inflammation. They showed that FHC was required to prevent sustained c-Jun N-terminal kinase cascade activation, thus inhibiting apoptosis induced by TNF-α. FHC-driven inhibition of c-Jun N-terminal kinase signaling depends on suppressing ROS generation and is achieved through kllits ability to sequester iron. Our results showed HCC cells over-expressing FHC generated less ROS when challenged by TNF-α and were resistant to apoptosis induced by serum starvation. These results suggested that autophagy conferred the TNF-α protection against starvation-mediated apoptosis of hepatocellular carcinoma cells, the process involved with transactivation of NF-κB, up-regulation of anti-apoptotic FHC, reactive oxygen species and caspase suppression. Understanding the contribution of TNF-α-mediated cell survival may be relevant to the therapy of hepatocellular carcinoma.

## Conclusion

Our findings suggested that autophagy conferred the TNF-α protection against serum starvation-mediated apoptosis of hepatocellular carcinoma cells, the process involved with transactivation of NF-κB, up-regulation of anti-apoptotic FHC, suppression of reactive oxygen species and caspase.

## Abbreviations

TNF-α: Tumor necrosis factor-α; NF-κB: Nuclear factor-κB; FHC: Ferritin heavy chain; ROS: Reactive oxygen species; DAPI: 4′,6′-diamidino-2-phenylindole dihydrochloride; DMEM: Dulbecco’s modified eagle medium; FBS: Fetal bovine serum; HCC: Hepatocellular carcinoma; 3-MA: 3-methyladenine; PBS: Phosphate buffered saline; RT-PCR: Reverse transcription polymerase chain reaction; NAC: N-acetyl Cysteine; IKK: I-κB kinase.

## Competing interests

The authors declare that they have no competing interests.

## Authors’ contributions

XRK, YYJ, WJD and LXW participated in the design and performance of the study. KS, ZPH and FY performed statistical analysis. GFY, QMF, LG, XZ, QDZ and RL carried out cell culture and molecular studies. MCW and LXW conceived of the study and participated in its design and coordination. The manuscript was drafted by XRK, YYJ and WJD, and reviewed by all authors. All authors approved the final version of the manuscript to be published. All authors read and approved the final manuscript.

## Pre-publication history

The pre-publication history for this paper can be accessed here:

http://www.biomedcentral.com/1471-2407/13/438/prepub

## Supplementary Material

Additional file 1: Figure S1 Inhibition autophagy with shRNA not only attenuated TNF-α protection against serum starvation-mediated apoptosis but also decreased the activation of NF-κB. Hep3B and SMMC-7721 cells were treated with serum starvation, after 6 h cells were transfected with shRNA against the essential autophagy Beclin1. Then cells were treated with or without TNF-α (10 ng/ml) for 24 h under serum starvation condition. (A and B) Shown is a representative Western blot comparing Beclin1 expression with a control shRNA. The bottom panel is a GAPDH loading control. (C and D) Cell activity was determined by MTT analysis. (E and F) Apoptosis was measured by flow cytometry. (G and H) Cells were transduced using an NF-κB luciferase construct, then the cells were treated according to the previously described steps. At the end of the various treatment, firefly and renilla luciferase activities were assessed using a dual luciferase reporter gene assay kit. Data are presented as the mean ± SEM from three independent experiments. **P<0.01; Student’s t-test. **Figure S2**. Inhibition of NF-κB inhibited TNF-α protection and the FHC expression. Serum-deprived Hep3B and SMMC-7721 cells were cultured with TNF-α (10 ng/ml) and/or pBαbe/pBαbe-SR-IκBα. (A and B) Expression of IκBa protein following pBabe-SR-IκBa plasmid transfection, demonstrated by western blot analysis. (C and D) Cells were harvested at 24 h, FITC/PI staining was performed to detect the apoptosis. (E and F) The expression of FHC was determined by RT-PCR and Western-blotting analysis. Data are presented as the mean ± SEM from three independent experiments. **P<0.01; Student’s t-test. Click here for file
