# Supplementary material for: Tumor necrosis factor-α attenuates starvation-induced apoptosis through upregulation of ferritin heavy chain in hepatocellular carcinoma cells
Source: BMC Cancer. 2013 Sep 25;13:438. doi: 10.1186/1471-2407-13-438 (PMC3849379; doi:10.1186/1471-2407-13-438)
Supplement: Additional file 1: Figure S1 — Inhibition autophagy with shRNA not only attenuated TNF-α protection against serum starvation-mediated apoptosis but also decreased the activation of NF-κB. Hep3B and SMMC-7721 cells were treated with serum starvation, after 6 h cells were transfected with shRNA against the essential autophagy Beclin1. Then cells were treated with or without TNF-α (10 ng/ml) for 24 h under serum starvation condition. (A and B) Shown is a representative Western blot comparing Beclin1 expression with a control shRNA. The bottom panel is a GAPDH loading control. (C and D) Cell activity was determined by MTT analysis. (E and F) Apoptosis was measured by flow cytometry. (G and H) Cells were transduced using an NF-κB luciferase construct, then the cells were treated according to the previously described steps. At the end of the various treatment, firefly and renilla luciferase activities were assessed using a dual luciferase reporter gene assay kit. Data are presented as the mean ± SEM from three independent experiments. **P<0.01; Student’s t-test. Figure S2. Inhibition of NF-κB inhibited TNF-α protection and the FHC expression. Serum-deprived Hep3B and SMMC-7721 cells were cultured with TNF-α (10 ng/ml) and/or pBαbe/pBαbe-SR-IκBα. (A and B) Expression of IκBa protein following pBabe-SR-IκBa plasmid transfection, demonstrated by western blot analysis. (C and D) Cells were harvested at 24 h, FITC/PI staining was performed to detect the apoptosis. (E and F) The expression of FHC was determined by RT-PCR and Western-blotting analysis. Data are presented as the mean ± SEM from three independent experiments. **P<0.01; Student’s t-test. [file 1471-2407-13-438-S1.doc]

**Supplemental Figure legends**

**Supplementary Figure.1 Inhibition autophagy with shRNA not only attenuated TNF-α protection against serum starvation-mediated apoptosis but also decreased the activation of NF-κB.** Hep3B and SMMC-7721 cells were treated with serum starvation, after 6 h cells were transfected with shRNA against the essential autophagy Beclin1. Then cells were treated with or without TNF-α (10 ng/ml) for 24h under serum starvation condition. (A and B) Shown is a representative Western blot comparing Beclin1 expression with a control shRNA. The bottom panel is a GAPDH loading control. (C and D) Cell activity was determined by MTT analysis. (E and F) Apoptosis was measured by flow cytometry. (G and H) Cells were transduced using an NF-κB luciferase construct, then the cells were treated according to the previously described steps. At the end of the various treatment, firefly and renilla luciferase activities were assessed using a dual luciferase reporter gene assay kit. Data are presented as the mean ± SEM from three independent experiments. **P<0.01; Student’s t-test.

**Supplementary Figure.2 Inhibition of NF-κB inhibited TNF-α protection and the FHC expression.** Serum-deprived Hep3B and SMMC-7721 cells were cultured with TNF-α (10 ng/ml) and/or pBαbe/pBαbe-SR-IκBα. (A and B) Expression of IκBa protein following pBabe-SR-IκBa plasmid transfection, demonstrated by western blot analysis. (C and D) Cells were harvested at 24h, FITC/PI staining was performed to detect the apoptosis. (E and F) The expression of FHC was determined by RT-PCR and Western-blotting analysis. Data are presented as the mean ± SEM from three independent experiments. **P<0.01; Student’s t-test.
